# Comprehensive evaluation of chemiluminescent immunoassays for the laboratory diagnosis of *Clostridium difficile* infection

**DOI:** 10.1007/s10096-017-2916-9

**Published:** 2017-02-08

**Authors:** A. Makristathis, I. Zeller, D. Mitteregger, M. Kundi, A. M. Hirschl

**Affiliations:** 1 Division of Clinical Microbiology, Department of Laboratory Medicine, Medical University Vienna, Vienna, Austria; 2 Department of Environmental Hygiene, Medical University Vienna, Vienna, Austria

## Abstract

For the microbiological diagnosis of a *Clostridium (C.) difficile* infection (CDI), a two-test algorithm consisting of a *C. difficile* glutamate dehydrogenase (GDH)-immunoassay followed by a toxin-immunoassay in positive cases is widely used. In this study, two chemiluminescent immunoassays (CLIAs), one for GDH and the other for the toxins A and B, have been evaluated systematically using appropriate reference methods. Three-hundred diarrhoeal stool specimens submitted for CDI diagnosis were analysed by the LIAISON CLIAs (DiaSorin). Toxigenic culture (TC) and cell cytotoxicity assay (CCTA) were used as “gold standard” reference methods. In addition, GDH and toxin A and B enzyme immunoassays (EIAs), C. diff Chek-60 and toxin A/B II (TechLab), and the Cepheid Xpert *C. difficile* polymerase chain reaction (PCR) were performed. *C. difficile* was grown in 42 (14%), TC was positive in 35 (11.7%) and CCTA in 25 (8.3%) cases. CLIAs were more sensitive but less specific than the respective EIAs. Using culture as reference, the sensitivity of the GDH CLIA was 100%. In comparison to CCTA sensitivity, specificity, positive predictive value and negative predictive value of the two-test algorithm were 88, 99.3, 91.7 and 98.9% by CLIAs and 72, 99.6, 94.7 and 97.5% by EIAs. Discrepant results by CLIAs were more frequent than that by EIAs (9% vs. 6.3%); in those cases, PCR allowed for the accurate detection of toxigenic strains. Due to performance characteristics and testing comfort, CLIAs in combination with PCR represent a favourable option for the rapid laboratory *C. difficile* diagnostics.

## Introduction

*Clostridium (C.) difficile* infection (CDI) can cause a spectrum of diseases from self-limiting diarrhoea and pseudomembranous colitis (PMC) to life-threatening conditions such as toxic megacolon, perforation of the gut, and sepsis. CDI is a major cause of diarrhoea in healthcare facilities commonly affecting the most vulnerable patients after the use of antibiotics, but was also shown to account for approximately 2% of community acquired cases of diarrhoea [1, 2]. Toxigenic strains producing the toxins A and B are responsible for disease. During recent years, the incidence of hospital-acquired *C. difficile*-associated diarrhoea has increased in many countries, which may be due—at least in part—to the epidemic spread of hypervirulent strains (e.g. ribotype 027) [3, 4].

Accurate and rapid diagnosis of CDI is of crucial importance to ensure that patients receive appropriate treatment and to control the spread of the infection. Clinical and laboratory diagnoses are complicated by the fact that up to 25% of hospitalised patients may be colonised with toxigenic *C. difficile* strains without any symptoms [5]. Therefore, the detection of a toxigenic strain in the faeces of a symptomatic patient must not necessarily be the reason for the diarrhoea. Thus, the presence of *C. difficile* toxins in faeces should correlate better with CDI than culture of a toxigenic isolate (toxigenic culture, TC) [6].

The “gold standard” reference method for the detection of *C. difficile* toxins (mainly toxin B) in faeces is considered to be the cell cytotoxicity assay (CCTA) [7]. However, this is a lengthy assay with a time to result of 24 to 48 h. Alternatively, several commercially available enzyme immunoassays (EIAs) and lateral-flow assays for diagnosis of toxins A and B have been widely used. However, considerable differences were shown between those tests in their performance characteristics [8, 9].

Because of the low prevalence of CDI both in the healthcare and especially in the community setting the commercially available tests exhibit high negative predictive values (NPVs), but the positive predictive values (PPVs) are unacceptably low. This applies not only to toxin EIAs but also to those detecting the *C. difficile* surface-associated enzyme glutamate dehydrogenase (GDH) and nucleic acid amplification tests (NAATs) for the detection of either toxin A or B genes [8–12]. However, GDH EIAs and NAATs show satisfactory sensitivities in comparison to culture and TC, respectively [10–14].

While none of these tests is appropriate to be used as a stand-alone test, a two-step algorithm has been proposed by the UK Department of Health guidance. This algorithm consists of a GDH immunoassay or NAAT as screening test followed by a sensitive toxin immunoassay for the re-analysis of all positive samples. Due to considerably increased pre-test probability this approach would yield excellent PPVs and due to toxin detection it may also show a better correlation with the disease [6, 15–19].

During recent years, two chemiluminescent immunoassays (CLIAs), one for the detection of *C. difficile* GDH and the other for the detection of toxins A and B, became commercially available. The CLIA two-step algorithm allows for higher testing comfort and flexibility as well as shorter time-to-result in comparison to testing by EIAs. However, only few published data exist with these tests [20, 21], and a systematic evaluation of each of them with an appropriate reference method was not provided in all cases. In the present study, the performance of each of the CLIAs has been compared to an appropriate “gold standard” reference method as well as a widely used EIA, one for GDH and another for toxins A and B. Also the performance characteristics of two-step algorithms using the combination of either CLIAs or EIAs have been analysed in comparison to both TC and CCTA.

## Materials and methods

### Samples

Three hundred diarrhoeal stool specimens submitted for CDI diagnosis have been included consecutively, if they were less than 24 h old and of sufficient volume to allow testing with all study-related assays in addition to routine testing. The latter comprised an initial screening by a GDH EIA (C. diff Chek-60; TechLab, Blacksburg, VA, USA). In case of a positive result confirmatory PCR testing (Xpert *C. difficile* assay; Cepheid, Sunnyvale, CA, USA) was conducted. Specimens were given a study protocol number by the routine staff and were, thereby anonymised, forwarded to the scientific staff for further analysis by TC, CCTA, EIA for toxins A and B and the two CLIAs, one for GDH and the other for toxins A and B. The results of routine testing were communicated to the clinical ward; those obtained by the study-related tests were not disclosed, and therefore, did not impact the patient’s management.

### Cell cytotoxicity assay

Faecal samples were diluted with an equal volume of PBS, centrifuged and the supernatants were filtered through a 0.2 μm filter. The assay was performed by testing this sterile faecal filtrate in duplicates on a confluent monolayer of Vero cells after preincubation of cells with either PBS or *C. difficile* antitoxin. The final dilution of the stools was 1:40. For positive and neutralisation controls the *Clostridium difficile* toxin/antitoxin kit was used according to the instructions of the manufacturer (TechLab). A positive result was recorded if cell rounding was seen only in the unprotected cells after 24 or 48 h of incubation in a 37 °C CO_2_-incubator.

### Culture

All samples were cultured on *Clostridium difficile* agar (bioMérieux, Marcy l’Etoile, France), following an alcohol shock treatment in 50:50 [vol/vol] absolute ethanol and water. Cultures were incubated in an anaerobic atmosphere for 48 h. Suspicious colonies (gray-brown colonies with irregular edges and a characteristic horse manure like odour) were further analysed by matrix-assisted laser desorption ionisation - time of flight mass spectrometry (MALDI-TOF MS) on a Microflex instrument (Bruker Daltonics, Bremen, Germany).

### Toxigenic culture

*C. difficile* isolates were inoculated to brain heart infusion broth (BHI) (Oxoid, Basingstoke, United Kingdom) and incubated for 48 h in anaerobic jars. Culture supernatants were centrifuged before being added to Vero cell monolayers. For each sample, two wells were prepared (with and without *C. difficile* antitoxin).

### Commercial tests

C. diff Chek-60 and toxin A/B II, a GDH and a toxin EIA respectively, were performed as described by the manufacturer (TechLab) on a Tecan Minilyser instrument (Tecan Group Ltd., Männedorf, Switzerland). The chemiluminescent immunoassays LIAISON *C. difficile* GDH and LIAISON *C. difficile* Toxins A&B (DiaSorin Inc, Stillwater, MN, USA) were performed according to the manufacturers’ recommendations on a LIAISON Analyzer (DiaSorin). The Cepheid Xpert *C. difficile* assay, which is a multiplex real-time PCR assay, was performed on the Cepheid GeneXpert Dx system following the manufacturers’ instructions.

### Statistical analysis

Sensitivity, specificity, PPV and NPV and their 95% confidence intervals were calculated for each commercial kit against the appropriate reference assay. No statistical comparisons were performed between the toxin detection assays and the GDH assays, as these measure different targets. Sensitivity and specificity were compared by applying exact binomial tests on the off-diagonal counts of the McNemar table, PPV and NPV by Fisher’s exact probability test. A *p*-value below 0.05 was considered significant.

## Results

Out of 300 samples, *C. difficile* could be grown in 42 (14%) and TC was positive in 35 (11.7%) cases. Thus 83.3% of the isolates were toxigenic. CCTA was positive in 25/300 cases (8.3%), all of which were also positive by TC. Thus, CCTA was positive in 71.4% (25/35) of TC positive cases.

In comparison to culture, the sensitivity, specificity, PPV and NPV of the GDH testing were 88.1, 99.6, 97.4 and 98.1% by EIA and 100, 96.5, 82.4 and 100% by CLIA (Table 1). In one case culture, PCR and all toxin tests were negative, but both GDH immunoassays were positive; thus, it cannot be excluded that a non-toxigenic strain might have been present and the culture result was potentially false negative. Statistical analysis revealed that for the detection of GDH specificity and PPV were significantly higher by EIA in comparison to CLIA (*p* = 0.013 and *p* = 0.039, respectively), whereas, on the other hand, the NPV by CLIA was significantly higher than that by EIA (*p* = 0.029).

**Table 1:** GDH assays compared to culture

Assay	Finding	Culture		% Sensitivity (95% CI)	% Specificity (95% CI)	% PPV (95% CI)	% NPV (95% CI)
		Positive	Negative				
GDH EIA	Positive	37	1	88.1 (73.6–95.5)	99.6^a^ (97.5–100)	97.4^a^ (84,6–99.9)	98.1 (95.4–99.3)
	Negative	5	257				
GDH CLIA	Positive	42	9	100 (89.6–100)	96.5 (93.3–98.3)	82.4 (68.6–91.1)	100^b^ (98.1–100)
	Negative	0	249				

*CLIA* chemiluminescent immunoassays, *EIA* enzyme immunoassays, *CI* confidence interval, *PPV* positive predictive values, *NPV* negative predictive values

^a^Statistically significantly higher than by CLIA

^b^Statistically significantly higher than by EIA

Sensitivity, specificity, PPV and NPV of the toxin assays in comparison to CCTA were 72.0, 97.5, 72.0 and 97.5% by EIA and 88.0, 94.9, 61.1 and 98.9% by CLIA (Table 2). Similarly to the results for GDH testing, the toxin CLIA showed higher sensitivity and lower specificity when compared to the toxin EIA. However, the differences were not of statistical significance. In one case CCTA was most likely false negative because all other tests, including both toxin immunoassays, were positive.

**Table 2:** Toxin A&B assays compared to cell cytotoxicity assay

Assay	Finding	Cell cytotoxicity assay		% Sensitivity (95% CI)	% Specificity (95% CI)	% PPV (95% CI)	% NPV (95% CI)
		Positive	Negative				
Toxin EIA	Positive	18	7	72.0 (50.6–87.9)	97.5 (84.8–99.0)	72.0 (50.6–87.9)	97.5 (84.8–99.0)
	Negative	7	268				
Toxin CLIA	Positive	22	14	88.0 (68.8–97.5)	94.9 (91.6–97.2)	61.1 (43.5–76.9)	98.9 (96.7–99.8)
	Negative	3	261				

*CLIA* chemiluminescent immunoassays, *EIA* enzyme immunoassays, *CI* confidence interval, *PPV* positive predictive values, *NPV* negative predictive values

In comparison to TC the sensitivity, specificity, PPV and NPV of the two-step algorithms were 54.3, 100, 100 and 94.3% by EIAs and 68.6, 100, 100 and 96.0% by CLIAs (Table 3). In comparison to CCTA the respective values were 72, 99.6, 94.7 and 97.5% by EIAs and 88, 99.3, 91.7 and 98.9% by CLIAs (Table 4). Irrespective of the GDH test used for the two-step comparisons to TC or CCTA, values in the *χ* ^2^ test (Tables 3 and 4) were dependent solely on the toxin test. Thus, numbers with the two EIAs were exactly the same as those of the combination of the GDH CLIA with the toxin EIA, and numbers with the two CLIAs were identical to those of the combination of the GDH EIA with the toxin CLIA.

**Table 3:** GDH/toxin A&B algorithm by EIAs and CLIAs with and w/o PCR compared to toxigenic culture

Assay	Finding	Toxigenic culture		% Sensitivity (95% CI)	% Specificity (95% CI)	% PPV (95% CI)	% NPV (95% CI)
		Positive	Negative				
EIAs	Positive^a^	19	0	54.3 (36.7–71.2)	100 (98.6–100)	100 (82.4–100)	94.3 (90.9–96.7)
	Negative^b^	16	265				
EIAs + PCR^c^	Positive^d^	33	0	94.5^f^ (80.8–99.3)	100 (98.6–100)	100 (89.4–100)	99.3^f^ (97.3–99.9)
	Negative	2^e^	265				
CLIAs	Positive^a^	24	0	68.6 (50.7–83.2)	100 (98.6–100)	100 (85.8–100)	96.0 (93.0–98.0)
	Negative^b^	11	265				
CLIAs + PCR^c^	Positive^d^	35	0	100^f^ (90.0–100)	100 (98.6–100)	100 (90.0–100)	100^f^ (98.6–100)
	Negative	0	265				

*CLIA* chemiluminescent immunoassays, *EIA* enzyme immunoassays, *CI* confidence interval, *PCR* polymerase chain reaction, *PPV* positive predictive values, *NPV* negative predictive values

^a^Positive GDH assay confirmed by the toxin assay

^b^Either one of the assays or both assays negative

^c^PCR performed only in discrepant cases

^d^Either both immunoassays positive or positive PCR result in GDH positive and toxin negative cases

^e^False negative GDH assay

^f^Statistically significantly higher than by the respective two-step algorithm

**Table 4:** GDH/toxin A&B algorithm by EIAs and CLIAs compared to cell cytotoxicity assay

Assay	Finding	Cell cytotoxicity assay		% Sensitivity (95% CI)	% Specificity (95% CI)	% PPV (95% CI)	% NPV (95% CI)
		Positive	Negative				
EIAs	Positive^a^	18	1	72.0 (50.4–87.1)	99.6 (97.7–100)	94.7 (71.9–99.7)	97.5 (94.7–98.9)
	Negative^b^	7^c^	274				
CLIAs	Positive^a^	22	2	88.0 (67.7–96.8)	99.3 (97.1–99.9)	91.7 (71.5–98.5)	98.9 (96.6–99.7)
	Negative^b^	3^c^	273				

*CLIA* chemiluminescent immunoassays, *EIA* enzyme immunoassays, *CI* confidence interval, *PPV* positive predictive values, *NPV* negative predictive values

^a^Positive GDH assay confirmed by the toxin assay

^b^Either one of the assays or both assays negative

^c^True positive GDH assay and negative toxin assay

In 19 cases (6.3%) the EIA GDH test was positive whereas the toxin test was negative. In 7/19 cases TC, PCR and CCTA were positive. Of the remaining 12 samples, seven were positive by TC and PCR and four were positive by culture; the one which was positive solely by both GDH immunoassays was considered false positive. As with CLIAs, in 27 cases (9%) the GDH test was positive and the toxin test was negative. Of those, in three, TC, PCR and CCTA were positive; in eight, both TC and PCR were positive; in seven, culture was positive; and in nine, the GDH test was considered false positive. Conversely, in six samples by EIAs and 12 by CLIAs we observed a positive toxin result although the GDH test was negative; all of these toxin test results were considered false positive. In accordance with the performance characteristics of the two GDH and the two CLIA assays, the lowest number of discrepant cases (*n* = 14) was observed by the combination of the GDH EIA with the toxin CLIA and the highest (*n* = 32) by the combination of the GDH CLIA with the toxin EIA.

PCR was performed in all GDH EIA positive cases but also, in retrospect, in the two TC positive/GDH EIA negative cases (Table 3) always yielding a positive result. Thus, the sensitivity of PCR in comparison to TC was shown to be 100%. Performing PCR in discrepant cases either by EIAs or CLIAs would improve the sensitivity of the respective two-step algorithm towards TC in a highly significant manner (*p* < 0.001; Table 3). Assay combinations including PCR were dependent solely on the GDH assay irrespective of the toxin test used. Thus, in a three-step algorithm the combination of the GDH CLIA with the toxin EIA would result in the same outcome as that of the two CLIAs, and the combination of the GDH EIA with the toxin CLIA would result in the same values as those obtained by the two EIAs (Table 3).

## Discussion

The use of CLIAs in *C. difficile* diagnosis represents an alternative to EIAs. The two EIAs used in the present study–C. diff Chek-60 and toxin A/B II–have been evaluated in a number of studies and are well-established for routine use [6, 8–11, 18, 19]. The comparison between the GDH assays using culture as reference revealed a lower specificity and PPV but an excellent sensitivity and NPV of the CLIA, which was also shown by others [20]. Thus, all culture-positive cases could be detected by CLIA which suggests the appropriateness of this test for the screening of CDI in stool samples.

With regard to the toxin assays, the higher sensitivity of CLIA (88.0% vs. 72.0% when compared to the EIA) may also disclose the suitability of this assay for toxin detection in GDH antigen positive cases. However, analogue to the GDH assays, the toxin CLIA was less specific than the toxin EIA.

The combination of the GDH with the toxin CLIA, both showing higher sensitivities than the respective EIAs, resulted in a considerably higher overall sensitivity than that shown by the EIA testing algorithm. Thus, in comparison to CCTA, which was recently shown to be the appropriate reference method for the laboratory confirmation of CDI [6, 15], the difference in sensitivity of the two-step algorithm between CLIAs and EIAs was 16%. Despite lower specificity values of both CLIAs in comparison to the respective EIAs, the specificity of the combined testing in comparison to TC was 100%. There were only two cases which were considered false positive by the CLIA two-step algorithm in comparison to CCTA (Table 4). However, in one of those cases all other tests including both toxin immunoassays were positive which suggests a false negative CCTA result. In the second case a toxigenic strain has been identified by TC and PCR, but in contrast to the CCTA and toxin EIA only the toxin CLIA yielded a positive result marginally above the threshold value. Thus, even in that case a low toxin concentration—not detected by the CCTA—may have been present.

A considerable proportion of cases with positive GDH but negative toxin CLIA results were attributable to a false positive result by the GDH assay (9/27, 33.3%). Quite contrary to CLIA testing, a similarly high proportion of such discrepant cases found by EIAs was due to false negative results by the toxin assay (7/19, 36.8%). The proportion of cases of absence of toxins or presence of non-toxigenic strains leading to positive GDH and negative toxin assay results was similar between discrepant cases found by CLIAs and EIAs (15/27, 55.6% vs. 11/19, 57.9%). Overall, the number of discrepant results by CLIAs was higher than that by EIAs.

For further clarification of cases with positive GDH and negative toxin test results it would be reasonable to introduce a rapid NAAT as a third test. In this and previous studies the Cepheid Xpert *C. difficile* PCR showed an excellent performance in comparison to TC. Some authors even suggested using this PCR as a single laboratory test for CDI diagnosis [12, 14, 22–24]. However, apart from a considerable cost, the appropriateness of NAATs for the laboratory confirmation of CDI has been a controversial issue in recent literature [6, 15, 25]. Nonetheless, irrespective of the clinical relevance of a positive molecular test result, it definitely identifies the presence of a toxigenic strain, which may influence measures of infection control.

In the present study, PCR results, being available as part of the routine analysis, were in total agreement with TC. PCR was positive in the majority of cases (14/19, 73.7%) of positive GDH and negative toxin test results by EIAs, but only in 40.7% (11/27) of such discrepant cases by CLIAs. However, irrespective of the test system used, CLIAs or EIAs, the implementation of PCR as a third test should be seriously considered, because this would allow for the accurate detection of toxigenic strains among discrepant cases. In the present study, the three-step algorithm would result in significantly higher sensitivities both by CLIAs and EIAs (100% vs. 68.6% and 94.5% vs. 54.3%, respectively; *p* < 0.001) in comparison to TC.

Apart from diagnostic performance characteristics issues, CLIAs showed to be more practical and convenient than the EIAs for routine use. In contrast to Benedek et al. [20], we did not experience any noteworthy technical drawbacks with the system, upon optimization of the instrument settings of the LIAISON Analyzer. Samples can be processed individually upon their arrival without the need to collect samples prior to testing. The sample preparation time is less than 15 min with only a few minutes of actual hands-on time. If the GDH test is positive, the toxin assay is performed automatically without the need for manual intervention. The time-to-result is less than an hour for the GDH test and takes a further 45 min when a positive GDH result is followed by toxin testing. In case of a discrepant result between GDH and toxin assays, a rapid NAAT, e.g. the Cepheid Xpert *C. difficile* PCR, can easily be performed subsequently allowing for the generation of a same-day final report; due to the longer time-to-result of more than 2.5 h for subsequent testing and the lack of testing flexibility, this may not be as feasible by EIA testing, particularly when tests are performed manually.

The combination of the GDH EIA with the toxin CLIA may also be taken into consideration, since it was associated with the lowest number of discrepant results. Even if being less practical than CLIA testing, it may lead to a lower overall cost in case of a three-step algorithm including PCR.

In conclusion, CLIAs were more sensitive but less specific than the respective EIAs. In particular, the GDH CLIA with 100% sensitivity in comparison to culture clearly fulfils the requirements of a screening test in terms of excellent sensitivity. Moreover, the sensitivity of toxin detection in GDH positive cases in comparison to CCTA was considerably higher when using CLIAs instead of EIAs. However, the number of cases with positive GDH and negative toxin test results was higher by CLIAs than that by EIAs, suggesting a more urgent need of a third test, e.g. a rapid NAAT, for further clarification. Finally, due to their performance characteristics as well as the shorter hands-on time, shorter time-to-result and higher flexibility by testing in comparison to EIAs, CLIAs in combination with a NAAT may currently represent the most suitable option for rapid laboratory *C. difficile* diagnostics.
